# Perioperative blood transfusion adversely affects prognosis after resection of lung cancer: a systematic review and a meta-analysis

**DOI:** 10.1186/1471-2482-14-34

**Published:** 2014-05-23

**Authors:** Haixing Luan, Feng Ye, Lupeng Wu, Yanming Zhou, Jie Jiang

**Affiliations:** 1 Department of Anesthesiology, First Hospital Affiliated to Xiamen University, Xiamen 361003, China; 2 Department of Hepatobiliary & Pancreatovascular Surgery, Oncologic Center of Xiamen; First affiliated Hospital of Xiamen University, Xiamen 361003, China; 3 Department of Thoracic Surgery, First affiliated Hospital of Xiamen University; Oncologic Center of Xiamen, Xiamen 361003, China

**Keywords:** Lung cancer, Blood transfusion, Survival, Surgery, Meta-analysis

## Abstract

**Background:**

It is speculated that blood transfusion may induce adverse consequences after cancer surgery due to immunosuppression. This study was intended to assess the impact of perioperative blood transfusion on the prognosis of patients who underwent lung cancer resection.

**Methods:**

Eligible studies were identified through a computerized literature search. The pooled relative risk ratio (RR) with 95% confidence interval (CI) was calculated using Review Manager 5.1 Software.

**Results:**

Eighteen studies with a total of 5915 participants were included for this meta-analysis. Pooled analysis showed that perioperative blood transfusion was associated with worse overall survival (RR: 1.25, 95% CI: 1.13-1.38; *P* <0.001) and recurrence-free survival (RR: 1.42, 95% CI: 1.20-1.67; *P* <0.001) in patients with resected lung cancer.

**Conclusions:**

Perioperative blood transfusion appears be associated with a worse prognosis in patients undergoing lung cancer resection. These data highlight the importance of minimizing blood transfusion during surgery.

## Background

Lung cancer is one of the most common cancers worldwide. Surgical resection is the most effective and potentially curative therapeutic option for this disease. Despite improvements in surgical and anesthetic techniques, a great number of patients need perioperative blood transfusions. The immunosuppression from blood products has led to concerns about its effects on the postoperative outcome of surgical oncology patients [1]. Some reports suggested that perioperative blood transfusion was associated with worse long-term oncological outcomes after surgery for lung cancer [2-5], but other studies failed to find such an association [6-9].

In the light of these conflicting findings, we performed a meta-analysis to elucidate the correlation between perioperative blood transfusion and prognosis in patients undergoing lung cancer resection.

## Methods

The study was conducted following the Preferred Reporting Items for Systematic Reviews and Meta- Analyses (PRISMA) [10].

### Literature search

A computerized search of the literature was performed by searching Medline, EMBASE, OVID, Cochrane database, and China National Knowledge Infrastructure from the time of inception to December 2013. The following medical subject heading terms were used: “lung cancer,” “blood transfusion,” “prognosis,” and “survival”. Only studies on humans and in the Chinese and English languages were eligible for inclusion. Reference lists of all identified articles were manually searched for additional studies.

### Inclusion and exclusion criteria

Inclusion criteria for primary studies were as follows: (i) the correlation between perioperative allogenenic blood transfusion and prognosis in patients undergoing lung cancer resection; and (ii) data available on overall survival (OS) or recurrence-free survival (RFS) with a median follow-up of at least 24 months. For duplicate publications reported by the same authors, either the one of higher quality or the most recent publication was selected. Abstracts, letters, editorials, expert opinions and reviews without original data were excluded from analysis.

### Data extraction

Two reviewers (LW and HL) independently extracted the following parameters from each study: first author, year of publication, country of origin, study population characteristics, study design, inclusion and exclusion criteria, numbers of participants, relative risk ratio (RR) or hazard ratio (HR) with 95% confidence interval (CI) for OS and RFS. All relevant texts, tables and figures were reviewed for data extraction. If additional data were needed, the authors were contacted to provide full details.

The quality of each included study was assessed using the Newcastle-Ottawa Scale consisting of three factors: patient selection, comparability of the study groups, and outcome assessment [11]. Studies achieving 6 or more stars were considered to be of higher quality.

### Outcome measurement

The primary outcomes of this study were OS and RFS.

### Statistical analysis and synthesis

The RR with 95% CI was used to evaluate the association between perioperative blood transfusions and RFS or OS. To do this, the HR was directly considered as RR. DerSimonian-Laird random-effect model was used to calculate the overall effect estimates. The RR was transformed to a natural log scale and then calculated for standard errors (SEs). Where HR was not reported, published data and figures from original papers were used to calculate the HR according to the methods described by Parmar *et al*. [12]. Heterogeneity across studies was evaluated with I^2^ statistics, with values up to 25%, 25%–50%, and above 50% indicating low, moderate, and high levels of heterogeneity. The RR was calculated by a random-effects model when the *P* value was less than 0.1. Otherwise, a fixed-effects model was used. Examination of publication bias was performed using a funnel plot based on the primary outcome. Sensitivity analyses were carried out by using the following subgroups: (i) studies of high quality; (ii) studies of patients with stage I disease; and (iii) studies containing more than 200 patients. All analyses were performed using the statistical software Review Manager version 5.1 (The Cochrane Collaboration, Software Update, Oxford).

## Results

### Eligible studies

We identified 647 potentially relevant records. After excluding studies that did not fulfill our inclusion criteria, 18 studies with a total of 5915 participants were included in the final meta-analysis [2,3,5-9,13-23]. The main features of the included studies are summarized in Table 1. Of these studies, eight studies were conducted in the USA [2,3,6-8,13,22,23], two in Italy [6,17], one in Finland [9], one in Poland [15], one in the United Kingdom [16], one in Spain [18], one in China [19], one in France [20], and one in Greece [21]. The number of patients ranged from 105 to 636 in each study. The transfusion rate in these reports ranged from 9.4 to 55.4%.

**Table 1:** Summary of studies included in the meta-analysis

**Reference (Year)**	**EI (Country)**	**FD (MM) (No. of lost)**	**Group**	**No. of patients (M/F)**	**Age, years**	**PHB, g/dl**	**Pathology Aa/Sc/Lc/Ot**	**TS I/II/II/IV**	**OT Pr/Pn**	**Quality score**
Tartter [2] (1984)	1966–1980 (USA)	24	Transfused	92 (−)	–	–	–	All stage I	–	4
		(−)	Non-transfused	73 (−)	–	–	–	All stage I	–	
Hyman [3] (1985)	1971–1979 (USA)	–	Transfused	33 (25/8)	59.5 ± 8	–	9/21/3/0	27/6/0/0	22/11	5
		(−)	Non-transfused	72 (58/14)	62.4 ± 7.1	–	20/38/11/3	67/5/0/0	55/17	
Pastorino [6] (1986)	1974–1979 (Italy)	–	Transfused	157 (147/10)	> 60, n = 75	–	53/79/25/0	All stage I	113/44	6
		(−)	Non-transfused	126 (117/9)	> 60, n = 64	–	49/59/18	All stage I	105/21	
Keller [7] (1988)	1974–1981 (USA)	(−)	Transfused	144 (91/53)	≥ 65.1, n = 73	–	–	119/25/0/0	127/17	5
		–	Non-transfused	208 (149/59)	≥ 65.1, n = 65	–	–	186/22/0/0	201/7	
Little [5] (1990)	1977–1986 (USA)	47	Transfused	58 (32/26)	61.3 ± 8.8	–	26/25/7/0	All stage I	48/10	5
		(0)	Non-transfused	59 (28/31)	60.1 ± 9.6	–	37/16/6/0	All stage I	51/8	
Pena [8] (1992)	1980–1984 (USA)	–	Transfused	30 (24/6)	66.1 ± 7.6	12.8 ± 1.9	10/15/3/2	23/7/0/0	22/8	6
		(−)	Non-transfused	97 (78/19)	62.0 ± 8.0	14.2 ± 1.3	41/41/12/3	75/22/0/0	71/26	
Piantadosi [13] (1994)	1974–1981 (USA)	43.2	Transfused	169 (−)	–	–	–	–	–	6
		(−)	Non-transfused	161 (−)	–	–	–	–	–	
Rainio [9] (1996)	1978–1980 (Finland)	–	Transfused	95 (88/7)	–	–	–	60/8/27/0	–	5
		(−)	Non-transfused	113 (102/11)	–	–	–	83/10/20	–	
Nosotti [14] (2003)	1995–2000 (Italy)	34	Transfused	69 (52/17)	65.6 ± 9.4	12.5 ± 1.2	39/28/2/0	All stage I	–	6
		(38)	Non-transfused	212 (153/59)	64.5 ± 9.5	13.3 ± 3.1	139/66/7/0	All stage I	–	
Rzyman [15] (2003)	1993–1997 (Poland)	46	Transfused	185 (155/30)	59.5	≤12, 25%	51/113/19/2	62/41/80/2	113/72	4
		(0)	Non-transfused	163 (125/38)	60.3	≤12, 12%	43/108/9/3	85/33/41/4	121/42	
Ghosh [16] (2004)	1996–2003 (UK)	23.2^#^	Transfused	120 (73/47)	72	–	62/58/0/0	29/66/27/0	All Pr	5
		(−)	Non-transfused	209 (99/110)	69	–	83/126/0/0	53/85/58/0	All Pr	
Berardi [17] (2005)	1996–2001 (Italy)	27	Transfused	97 (−)	–	–	–	–	–	4
		(−)	Non-transfused	342 (−)	–	–	–	–	–	
Peñalver [18] (2005)	1969-2000 (Spain)	208.6^#^	Transfused	125 (120/5)	62.6 ± 9.01	–	21/89/15/0	All stage I	81/44	5
		(−)	Non-transfused	731 (688/43)	61.9 ± 8.95	–	199/466/66/0	All stage I	584/147	
Chen [19] (2007)	1993-2002 (China)	–	Transfused	135 (110/25)	58.6 ± 11.2	13.8 ± 2.1	60/55/5/15	50/27/58/0	–	5
		(0)	Non-transfused	145 (117/28)	59.8 ± 11.1	14.2 ± 1.4	75/52/5/13	74/26/45/0	–	
Thomas [20] (2007)	1993–2002 (France)	–	Transfused	139 (−)	–	–	39/77/17/6	–	All Pn	5
		–	Non-transfused	228 (−)	–	–	70/113/25/20	–	All Pn	
Panagopoulos [21] (2008)	1999–2005 (Greece)	27.2	Transfused	85 (74/11)	64 ± 9	11.5 ± 1.6	30/46/7/2	33/27/23/2	45/40	4
		(0)	Non-transfused	246 (221/25)	64 ± 9	12.7 ± 1.3	85/132/24/5	115/65/61/5	164/82	
Ng [22] (2012)	2001–2009 (USA)	48	Transfused	63 (31/32)	74	12.6	43/12/6/2	All stage I	All Pr	6
		(0)	Non-transfused	298 (130/168)	67	13.5	187/69/21/21	All stage I	All Pr	
Cata [23] (2013)	2004–2006 (USA)	63.6	Transfused	60 (31/29)	66.2 ± 9.4	12.08 ± 1.58	–	23/16/21/0	–	6
		37	Non-transfused	576 (267/309)	65.1 ± 10.5	13.43 ± 1.42	–	328/115/131/0	–	

*EI* Enrolment interval; *UK* United Kingdom; *FD* Follow-up duration; *MM* Median months; *M* Male; *F* Female; *PHB* Preoperative hemoglobin; *TS* Tumor stage; *Aa* Adenocarcinoma; *Sc* Squamous cell carcinoma; *Lc* Large cell carcinoma; *Ot* Other types; *OT* Operation type; *Pr* Partial resection (segmentectomy; lobectomy; bilobectomy); *Pn* Pneumonectomy.

^#^Mean.

There was 100% agreement between the two reviewers.

### Primary outcomes

**Figure 1:**
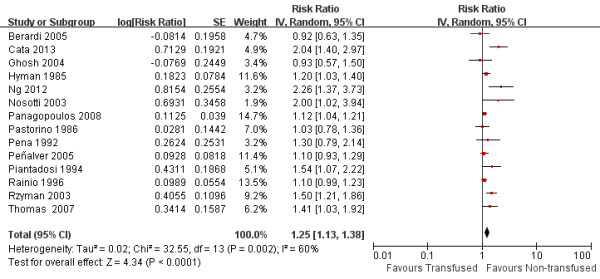
Forest plot showing the impact of perioperative blood transfusion on overall survival.

Data on OS were available from 14 studies. Univariate analysis alone was done in 2 [6,18] of the 14 studies. Multivariate analysis was done in the remaining 12 series [3,8,9,13-17,20-23]. In one study [15], the authors stated that there was no significant impact of transfusion in the multivariate analysis, but the statistic necessary for meta-analysis (RR, CI) was not reported; we therefore extracted the survival data from the Kaplan-Meier curve. The pooled data indicated that perioperative blood transfusion was associated with a worse OS (RR: 1.25, 95% CI: 1.13-1.38; *P* < 0.001) (Figure 1) in patients with resected lung cancer. As the test for heterogeneity was significant (I^2^ = 60%, *P* =0.002), a random-effects model was used to calculate the RR. Additional analyses in which the RR of the multivariate Cox model was pooled did not change the results significantly (RR: 1.27, 95% CI: 1.12-1.43; *P* < 0.001; I^2^ = 61%, *P* =0.004).

**Figure 2:**
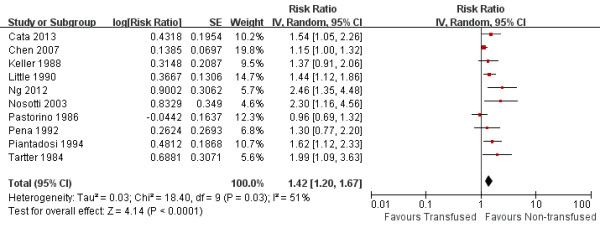
Forest plot showing the impact of perioperative blood transfusion on recurrence-free survival.

Data on DFS were available from10 studies. Univariate analysis alone was done in 1 [6] of the 10 studies. Multivariate analysis was done in the remaining 9 series [2,5-8,13,14,19,22,23]. In two studies [5,19], the authors stated that transfusion was an independent predictor of poor RFS, but the statistic necessary for meta-analysis (RR, CI) was not reported; we therefore extracted the survival data from the Kaplan-Meier curve. The pooled data indicated that perioperative blood transfusions was associated with a worse DFS (RR: 1.42, 95% CI: 1.20-1.67; *P* <0.001), with significantly heterogeneity between studies (I^2^ = 51%, *P* = 0.03) (Figure 2). Additional analyses in which the RR of the multivariate Cox model was pooled did not change the results significantly (RR: 1.64, 95% CI: 1.37-1.1.96; *P* <0.001; I^2^ = 0%, *P* =0.58).

### Sensitivity analysis

As Table 2 shows, the results derived from three subgroups were all consistent with those derived from overall meta-analysis.

**Table 2:** Results of sensitivity analysis

**Outcome**	**No. of studies**	**RR (95% CI)**	***P *** **value**	**I**^**2 **^**(%)**	**HG *p *** **value**
Patients with stage I disease					
OS	5 [6,14,18,21,22]	1.39 (1.03, 2.02)	0.02	66	0.02
RFS	5 [2,5-7,14,22]	1.51 (1.14, 2.01)	0.005	60	0.03
High-quality studies					
OS	6 [6,8,13,14,22,23]	1.58 (1.19, 2.08)	0.001	61	0.02
RFS	6 [6,8,13,14,22,23]	1.52 (1.14, 2.01)	0.004	57	0.04
Studies with >200 patients					
OS	12 [6,9,13-18,20-23]	1.26 (1.12, 1.41)	< 0.001	66	< 0.001
RFS	7 [6,7,13,14,19,22,23]	1.41 (1.13, 1.75)	0.002	61	0.02

*RR*, Relative risk ratio; *HG*, Heterogeneity; *OS*, Overall survival; *RFS*, Recurrence-free survival.

### Publication bias

**Figure 3:**
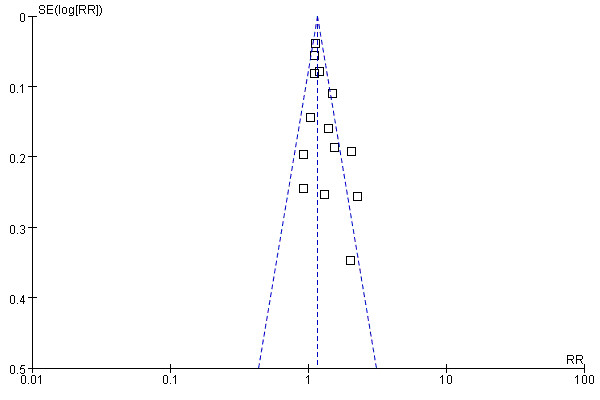
**Funnel plot analysis of publication bias.** The outcome was overall survival.

Visual assessment of a funnel plot of the studies used in the meta-analysis reporting on OS is shown in Figure 3. Two of the studies lay outside the limits of the 95% CI, indicating evidence of publication bias.

## Discussion

Blood transfusion is life saving in many circumstances but it also poses significant adverse effects, including incompatibility, transmission of viral diseases, coagulopathy, and allergic reactions [1]. In addition, it confers a significant cost and is an increasingly pressured resource. In 1982, Burrows and Tartter reported a higher recurrence rate in transfused patients after colon cancer resection as compared with matched untransfused patients [24]. Since then, numerous studies have addressed the effect of perioperative blood transfusion on patient survival after cancer surgery. Chung *et al*. [25] reviewed 20 studies that examined the effect of blood transfusion on prognosis after resection for colorectal carcinoma and found that transfusion was associated with an increased risk of tumor recurrence and cancer-related death. Also, in the field of hepatocellular carcinoma surgery, a recent meta-analysis conducted by Liu *et al*. [26] compared 22 studies that included 5635 patients and demonstrated that perioperative blood transfusion was associated with adverse clinical outcomes, including increased deaths, recurrences and complications. For lung cancer surgery, this subject is particularly relevant because of high transfusion rates ranging from 9.4% to 55.4%, as demonstrated in the present study. To the best of our knowledge, our study provides the first meta-analysis on the effect of perioperative blood transfusion on long-term outcomes after lung cancer surgery, for it included 18 studies with a sufficiently large sample size (n = 5915). The results show that perioperative blood transfusion has an unfavorable impact on prognosis in terms of OS and RFS.

Consistent with the clinical observations, experimental animal data indicate that blood transfusion facilitates tumor growth [27]. The most popular hypothesis is that blood transfusion-associated immunosuppressive alterations, such as the decreased helper/suppressor T-lymphocyte ratio, decreased natural killer cell function, defective antigen presentation and decreased cell-mediated immunity, might decrease tumor surveillance and worsen the prognosis [1]. In addition, there is evidence that transfusion has a significant impact on postoperative morbidity. In a retrospective analysis of 432 patients undergoing pneumonectomy for thoracic malignancies, the incidence of infectious complications was 13.7% in transfused patients and 5.6% in non-transfused patients (*P* =0.004) [20]. Infection induces the release of cytokines and chemokines including tumor necrosis factor-alpha, interleukin 6, and interleukin 8, which have been proposed as mediators of cancer development [28].

With respect to colorectal liver metastasis, Stephenson *et al*. [29] reported that patients who received more than 11 units of blood had significantly shorter disease-free intervals and worse survival than those who received 3–10 units of blood after surgery. Of the included studies in the current analysis, Pastorino, Keller, Little, Nosotti and their colleagues noted that the number of units transfused did not affect the survival or recurrence-free survival [5-7,14]. In contrast, Cata *et al*. [23] found that the number of units transfused was a factor associated with worse RFS and OS. We were unable to examine whether there was a dose-dependent effect of transfusion on survival because the stratification for the amount of transfused blood was not always the same between these studies.

Several weaknesses of the present study should be taken into consideration in interpreting our results. First, all the included studies were retrospective and are therefore subject to inherent biases, although the results of pooled data of multivariate RRs are similar to the findings from overall analysis. Second, funnel plot analysis revealed the sign of publication bias, which may relate to only published studies included. Third, significantly heterogeneity was detected within primay outcomes. There are considerable disparities between the studies that might introduce heterogeneity, including variation in the preoperative status (such as the American Society of Anesthesiologist physical status, body mass index, comorbidities and hemoglobin level), disease stage, the extent of resection and transfusion policies. In addition, some patients received preoperative or postoperative chemotherapy, which might have influenced the outcome. Also, it should be noted that these studies were conducted over a 20-year period, improvements in operative techniques and anesthesiological management as well as perioperative care are strongly linked to the outcome after lung cancer surgery. In order to minimize this effect, the RR was calculated by a random-effects model. Finally, it has been suggested that pre-, intra-, and postoperative administration of blood would increase the likelihood of colorectal cancer recurrences by 50, 74 and 36%, respectively [30]. Unfortunately, no study available has reported the effect of the timing of transfusion on long-term survival or tumor recurrence after lung cancer resection.

Given a negative effect of transfusion on lung cancer survival, both surgeons and anesthesiologists should be more prudent in using perioperative blood transfusion. Cata *et al.*[31] proposed an patient blood management protocol that comprises three main components: (i) evaluating high-risk patients and optimizing erythrocyte mass and function for such patients; (ii) minimizing perioperative erythrocyte loss through blood-sparing surgical techniques, maintenance of normothermia, intraoperative cell salvage techniques when appropriate, use of antifibrinolytics when indicated, and optimized fluid therapy and haemodynamic control; and (iii) using patient-specific transfusion triggers to decide when administration of blood products is warranted.

## Conclusions

The current literature review suggests that perioperative blood transfusion appears to be associated with a worse prognosis in patients undergoing lung cancer resection, which highlights the importance of avoiding or minimizing blood transfusion.

## Abbreviations

PRISMA: Preferred reporting items for systematic reviews and meta- analyses; OS: Overall survival; RFS: Recurrence-free survival; RR: Relative risk ratio; HR: Hazard ratio; CI: Confidence interval; SEs: Standard errors.

## Competing interests

The authors declare that they have no competing interests.

## Authors’ contributions

YZ participated in the design and coordination of the study, carried out the critical appraisal of studies and wrote the manuscript. HL, LW, JJ, and FY developed the literature search, carried out the extraction of data, assisted in the critical appraisal of included studies and assisted in writing up. YZ carried out the statistical analysis of studies. All authors read and approved the final manuscript.

## Pre-publication history

The pre-publication history for this paper can be accessed here:

http://www.biomedcentral.com/1471-2482/14/34/prepub
